# Management of Pseudohypoparathyroidism Type 1a during Pregnancy and Labor: A Case Report

**DOI:** 10.1155/2012/629583

**Published:** 2012-06-24

**Authors:** Anju Singh, Neelam Agarwal, Seema Chopra, Pooja Sikka, Vanita Suri, Bhupesh Kumar, Pinaki Dutta

**Affiliations:** ^1^Department of Obstetrics and Gynecology, Post Graduate Institute of Medical Education and Research, Chandigarh, India; ^2^Department of Anaesthesia and Intensive Care, Post Graduate Institute of Medical Education and Research, Chandigarh, India; ^3^Department of Endocrinology, Post Graduate Institute of Medical Education and Research, Chandigarh, India

## Abstract

Pseudohypoparathyroidism is rare during pregnancy and poses multiple challenges related to its diagnosis and management during pregnancy. We hereby report a case of a young woman who was diagnosed to have type 1a pseudohypoparathyroidism. She was managed by multidisciplinary team and had good maternal and perinatal outcome. Management-related issues are discussed here in detail.

## 1. Introduction

Pregnancy is commonly associated with altered calcium metabolism. Pseudohypoparathyroidism (PsHP) is rare during pregnancy [1]. It poses multiple diagnostic and management-related challenges during pregnancy besides a more difficult aspect of genetic counseling. To the best of our knowledge there is scant literature available for type 1a PsHP associated with pregnancy. In fact there is no publication regarding such case in the last 10 years. Here we report a case of type 1a PsHP presented to us during pregnancy with hypocalcemia and history of seizures. We discuss the management issues in such a case during pregnancy and labour.

## 2. Case Report

A 25-year-old pregnant lady was referred to our antenatal clinic at 24 weeks of gestation for investigations. She had a history of focal seizure, carpopedal spasm, and lip smacking movement since 6 years of age, for which she did not seek any medical advice. This was her second pregnancy which she had conceived spontaneously. Previously she had spontaneous abortion in first trimester. So far she had been supervised at a primary health centre and was apparently uncomplicated. There was no seizure activity during this pregnancy. She achieved menarche at 14  years of age, and her menstrual cycles were regular with scanty flow. At the age of 16 years, she had recurrent seizures for which she was prescribed oral phenytoin with some relief in symptoms. She stopped medication after 3 years without medical consultation.

Her general physical examination revealed height of 145 cm (<3rd centile), round facies, and short right 4th metacarpal. Further detailed examination showed signs of latent tetany in the form of positive Chvostek's sign and Trousseau's sign. Abdominal examination revealed uterus of approximately 24 weeks of gestation. Blood investigation showed low serum calcium (7.8 mg/dL) with high serum phosphate (5.8 mg/dL). The blood parathormone (PTH) level was raised (196 pg/mL) together with high alkaline phosphatase (200 U/L) and high 25(OH) vitamin D (29.77) level. The blood thyroxin level was below normal range and was associated with high thyroid stimulating hormone level. EEG revealed generalized epileptiform discharge. Computerized tomographic examination of brain showed multiple calcifications in bilateral globus pallidus, caudate nucleus, thalamus, frontal region, parietal lobes, and cerebellar hemispheres. Ultrasonographic parameters for fetal growth were found adequate. She was diagnosed with pseudohypoparathyroidism 1a and prescribed oral calcium (2.5 gm/day) and active vitamin D (1 *μ*g/day) together with levothyroxine 125 *μ*g daily in empty stomach. She had spontaneous onset of labor at 40 weeks of gestation. Her serum calcium was found very low (1.52 mg/dL) but without any seizures or tetany. She was managed with calcium infusion. Augmentation of labour with oxytocin failed to progress, and emergency cesarean section was done under regional anesthesia for nonprogress of labour. She delivered a healthy baby with a birth weight of 3.2 kg with good Apgar score. Her intraoperative and postoperative course was uneventful. She was discharged in satisfactory condition on fourth postpartum day. 

## 3. Discussion

PsHP is a disorder of calcium metabolism resulting from renal resistance to PTH. It is characterized by low serum calcium with elevated serum phosphate and parathormone levels. PsHP type 1a also shows resistance to other peptide hormones like thyroid stimulating hormone, gonadotropins, and gluacgon besides PTH. PsHP type 1a presents with characteristic phenotype, collectively called Albright's hereditary osteodystrophy. The constellation of findings includes short stature, stocky habitus, round face, skeletal anomalies, heterotopic calcification, and developmental delay. Patients may develop symptoms of hypocalcemia like paresthesias, muscle cramps, tetany, carpopedal spasm, or seizure. Primary hypothyroidism occurs in most patients with type 1a PsHP. Women may have delayed puberty, oligomenorrhea, and infertility. Our patient had normal menarche but scanty flow. There are also increased risks of spontaneous abortion, preterm labor, still birth, and intrauterine growth retardation together with increased risk of tetany during labour. She also had similar morphologic features, history of abortion, and symptoms of hypocalcemia together with hypothyroidism corroborating with type 1a PsHP.

Intermittent normocalcemia may occur spontaneously in patients with PsHP [2]. Although the exact mechanism is unknown, this is possibly due to PTH-mediated bone resorption [3]. Calcium metabolism is subtly altered in pregnancy, in relation to albumin levels and increased levels of parathyroid hormone. The effects on calcium homeostasis in PsHP during pregnancy are less clear and have rarely been studied [4]. During pregnancy, fetus avidly draw calcium from maternal circulation via active placental transport predisposing mother to hypocalcemia and its sequel [5]. Most such patient, requires therapeutic calcium and vitamin D supplementation to avoid maternal symptoms while ensuring adequate calcium supply to the fetus. However, some patients may become normocalcemic during pregnancy without ingesting therapeutic calcium and vitamin D. The mechanism by which PsHP improves during pregnancy is unclear. It may include placental secretion of parathyroid hormone-related protein (PHTrP) and calcitriol whose level has been reported to double or triple during second and third trimester of pregnancy in a report of two cases [6].

In pregnancy with PsHP, serum calcium needs frequent monitoring. It should not be allowed to fall below 6.8 mg/dL (1.70 mmol/L) to avoid preterm labor or midtrimester abortion. Hyperventilation during labor may precipitate hypocalcemia and -tetany, thus needs to be discouraged. Labour analgesia may be of some help by giving pain relief. Thyroxin supplementation and frequent monitoring of thyroid function are needed to maintain euthyroid status during pregnancy so as to avoid side effects of hypothyroidism on fetus. Fetus may also develop transient secondary hyperparathyroidism, leading to skeletal demineralization, subperiosteal bone resorption, osteitis fibrosa cystica, and intrauterine fractures. The effect is usually transient, manifests as poor feeding and fractures usually resolved by 7-8 months of life. During lactation, the requirement of calcium changes and needs frequent monitoring. The calcitriol dose should be reduced during lactation. The goal of therapy is to maintain serum total and ionized calcium level within reference range while avoiding hypercalciuria and to suppress PTH level to normal. Rarely elevated PTH levels in PsHP may cause increased bone remodeling leading to hyperparathyroid bone disease. 

## 4. Conclusion

The management of PsHP in pregnancy is quite intriguing. It needs regular calcium monitoring throughout pregnancy, labor, and postpartum period together with therapeutic supplementation of calcium and vitamin D as needed. The aim is to keep serum calcium in low normal range as overzealous calcium and vitamin D supplementation can cause hypercalcemia, hypercalciuria, and ectopic calcification. Keeping patients euthyroid with thyroxin supplementation is important for normal fetal development.
